# Comparison of an AI-enabled single-lead ECG monitor and point-of-care testing for detecting intradialytic hypokalemia

**DOI:** 10.1080/0886022X.2026.2746815

**Published:** 2026-10-30

**Authors:** Wen-Fang Chiang, Po-Jen Hsiao, Kun-Lin Wu, Ruei-Lin Wang, Kun-Yi Lin, Chi-Ming Chu, Jenq-Shyong Chan

**Affiliations:** a Division of Nephrology, Department of Internal Medicine, Taoyuan Armed Forces General Hospital, Taoyuan, Taiwan; b Division of Nephrology, Department of Internal Medicine, Tri-Service General Hospital, National Defense Medical University, Taipei, Taiwan; c Department of Life Sciences, National Central University, Taoyuan, Taiwan; d Department of Orthopedic Surgery, Tri-Service General Hospital, National Defense Medical University, Taipei, Taiwan; e Division of Biostatistics and Medical Informatics, Department of Epidemiology, School of Public Health, National Defense Medical University, Taipei, Taiwan; f Graduate Institute of Medical Sciences, National Defense Medical University, Taipei, Taiwan

**Keywords:** Artificial intelligence, electrocardiography, hemodialysis, hypokalemia, machine learning, point-of-care

## Abstract

**Background:**

Intradialytic hypokalemia is an underrecognized complication of maintenance hemodialysis (MHD). While point-of-care (POC) testing offers rapid biochemical confirmation, it remains limited by its intermittent nature. Artificial intelligence (AI)-enabled electrocardiography (ECG) provides a potential noninvasive alternative for trend monitoring; however, its performance in MHD has not been compared with POC testing against a central laboratory standard.

**Methods:**

This single-center prospective study compared AI-ECG potassium estimation and POC testing during MHD. An algorithm was trained on ECG–potassium pairs from 45 patients (2021–2022) and tested in 29 patients (2023). Concurrent laboratory–POC–ECG pairs enabled direct comparison, using central laboratory assays as the reference standard.

**Results:**

The AI-enabled ECG model achieved high accuracy within the non-hypokalemic range (mean absolute error (MAE) of 0.479 mmol/L), although the predictive variance expanded during hypokalemia (MAE of 1.000 mmol/L). POC testing demonstrated the highest laboratory concordance (MAE 0.201 mmol/L; ρ = 0.977). With respect to hypokalemia screening, the AI-ECG model maintained good discriminatory performance (area under the curve (AUC) of 0.722), which was paired with a high sensitivity (77.9%) and a robust negative predictive value (83.2%). POC testing significantly outperformed the AI-ECG model in diagnostic discrimination (AUC 0.996 vs. 0.722; ΔAUC 0.274; *p* < 0.0001).

**Conclusions:**

The AI-enabled ECG model demonstrates preliminary screening potential for hypokalemia alongside reliable non-hypokalemic tracking. Although POC testing remains the superior modality for point-in-time analytical precision, AI-enabled ECG offers a noninvasive approach for intradialytic trend monitoring. However, its ability to improve clinical outcomes or safely reduce invasive testing requires formal prospective decision-impact studies.

## Introduction

In patients with end-stage renal disease (ESRD), potassium homeostasis is frequently disrupted because of impaired renal excretion. While hyperkalemia typically develops between hemodialysis (HD) sessions, the rapid removal of potassium during treatment can precipitously trigger intradialytic or postdialysis hypokalemia. Crucially, this complication remains profoundly underrecognized in routine clinical practice, despite its severe clinical significance in terms of precipitating muscle weakness, ventricular arrhythmias, and sudden cardiac death [1–3]. A rapid decrease in serum potassium concentrations during HD is independently associated with increases in both all-cause mortality and cardiovascular mortality, a phenomenon likely driven by acute electrophysiological instability and heightened arrhythmogenesis [4,5]. Consequently, there is an urgent need for individualized monitoring strategies and precise dialysate potassium selection to mitigate these hidden risks [6].

Traditionally, serum potassium concentrations are monitored *via* intermittent laboratory testing before and after HD sessions. While highly accurate, this conventional “snapshot” approach fails to capture the dynamic fluctuations occurring throughout the treatment session and provides no real-time feedback to the clinical team. Point-of-care (POC) testing offers a more rapid bedside alternative, but it remains inherently intermittent, requires repeated invasive blood sampling, and incurs cumulative operational costs [7,8]. Furthermore, frequent POC measurements are resource intensive, rely heavily on operator techniques, and remain subject to preanalytical variability [9,10]. Thus, a clear clinical gap persists for noninvasive monitoring solutions capable of estimating dynamic intradialytic potassium trends.

Hypokalemia induces characteristic electrocardiographic (ECG) changes, including flattened or inverted T waves, prominent U waves, ST-segment depression, and PR interval prolongation [11]. However, these classic macrolevel markers are often absent or subtle in a significant portion of the dialysis population, and manual ECG interpretation suffers from suboptimal sensitivity and high interobserver variability [12]. Recent advances in artificial intelligence (AI) and machine learning offer a promising path forward for near real-time potassium estimation [13–17]. By identifying subtle, subvisual ECG features linked to potassium fluctuations, AI algorithms can estimate serum concentrations with high accuracy. Integrating this technology into standard dialysis workflows could allow for early trend detection, enabling clinicians to proactively adjust dialysate prescriptions or clinical interventions in real time [18].

In this study, we hypothesized that a machine learning–based analysis of single-lead ECG signals could effectively screen for and rule out intradialytic hypokalemia. We aimed to train and test an AI model to noninvasively estimate serum potassium concentrations in patients receiving maintenance hemodialysis (MHD) and subsequently compare its diagnostic performance with that of POC testing against a central laboratory standard.

## Methods

### Subjects and study design

Between June 2021 and December 2023, we prospectively enrolled patients with ESRD who received in-center MHD at a regional teaching hospital. The study adhered to the Declaration of Helsinki and was formally approved by the Institutional Review Board of the Tri-Service General Hospital (TSGHIRB No.: C202005172), with all participants providing written informed consent prior to data collection. Eligible participants were adults aged 18 years or older who had been receiving thrice-weekly, 4-h bicarbonate-based HD for at least three months using high-flux dialyzers and Toray TR-8000 machines. To ensure optimal signal clarity and maintain a strict focus on metabolic-induced changes, we excluded patients with a history of myocardial infarction, coronary artery bypass grafting surgery, pacemaker implantation, or atrial fibrillation, as well as those taking medications known to significantly alter baseline cardiac conduction.

### Laboratory and POC potassium measurement

To ensure precise sampling and mitigate the risk of access recirculation, blood samples were collected according to a strict, standardized intradialytic protocol: The anticoagulant line was securely clamped, the dialysate flow was completely paused for 3 min, and the blood flow rate was reduced to 100 mL/min for 15 s immediately prior to the draw. Serum potassium concentrations were determined in the central laboratory using an automated chemistry analyzer (DxC 700 AU, Beckman Coulter), with pseudohyperkalemia ruled out through laboratory assessment of sample hemolysis. For parallel comparisons, POC potassium concentrations were measured simultaneously from heparinized whole blood samples using an arterial blood gas analyzer (pHOx Ultra, Nova Biomedical). For all subsequent diagnostic and statistical analyses, hypokalemia was defined as a central laboratory-verified serum potassium concentration of less than 3.5 mmol/L.

### ECG acquisition and signal processing

Single-lead ECG signals were continuously recorded using a portable patient monitor (IntelliVue MP5, Philips) from the initiation to the completion of each HD session. Digitized waveforms were transmitted *via* a DS-600 upload gateway (ACE Biotek, Taiwan) to a secure web server. To ensure signal quality and model reliability, ECG segments characterized by severe baseline wander, excessive muscle noise, or significant motion artifacts were algorithmically and visually excluded from subsequent analysis. The preprocessing pipeline utilized a bandpass filter to eliminate low-frequency baseline drift and fixed-frequency powerline interference, followed by a discrete wavelet transform-based denoising algorithm to suppress irregular high-frequency noise while preserving core cardiac morphology. For feature extraction, a window of 250 data points preceding and succeeding each detected R-peak was captured. To maintain spatial-temporal uniformity across the recordings, all the extracted segments were downsampled to a standardized frequency of 100 Hz. Guided by previous research suggesting that T-wave slope and amplitude are highly informative of potassium concentrations, we focused on these discrete morphological features to train our downstream machine learning classification and regression models [14]. Details are presented in Figures 1 and 2 and the Supplemental Materials.

**Figure 1.**
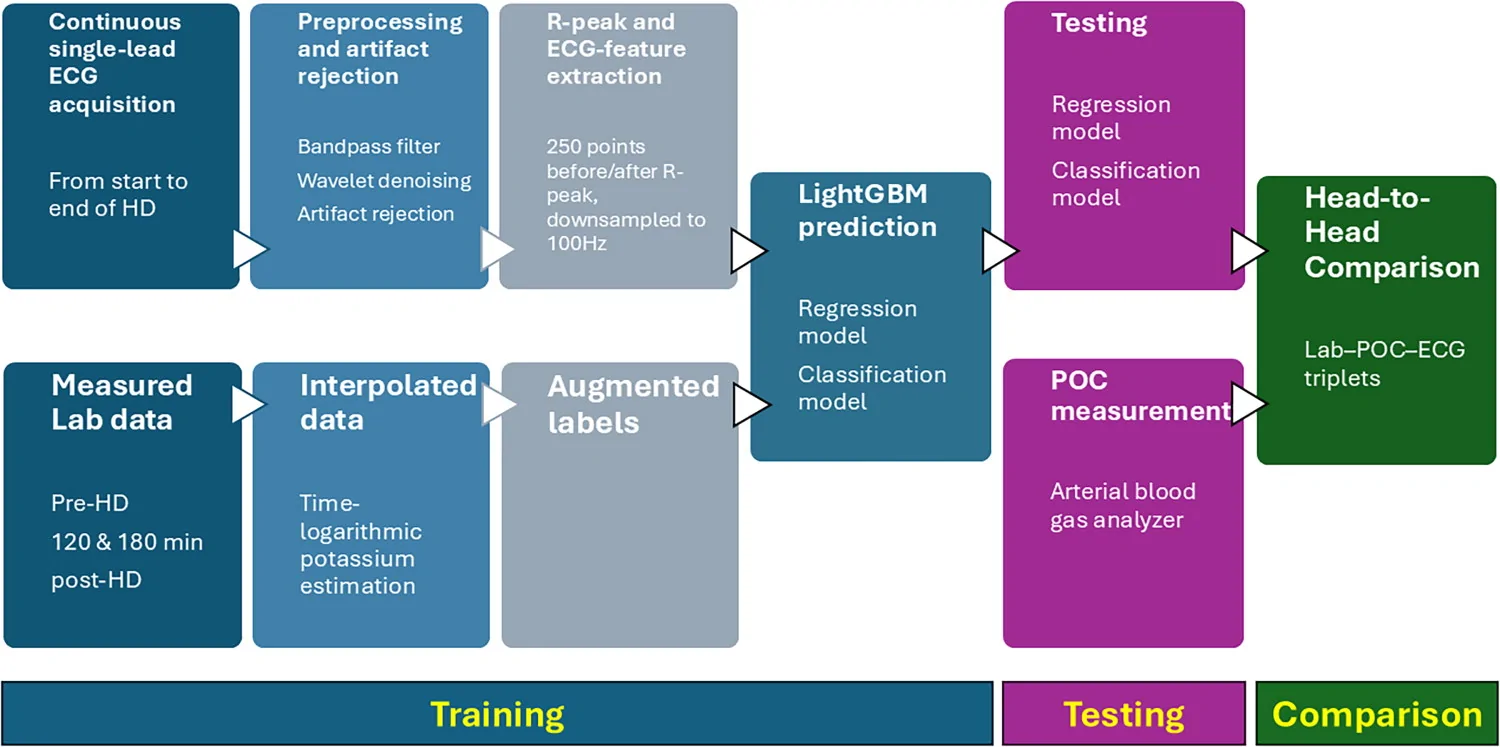
Methodological schematic of the AI-ECG potassium estimation framework.

**Figure 2.**
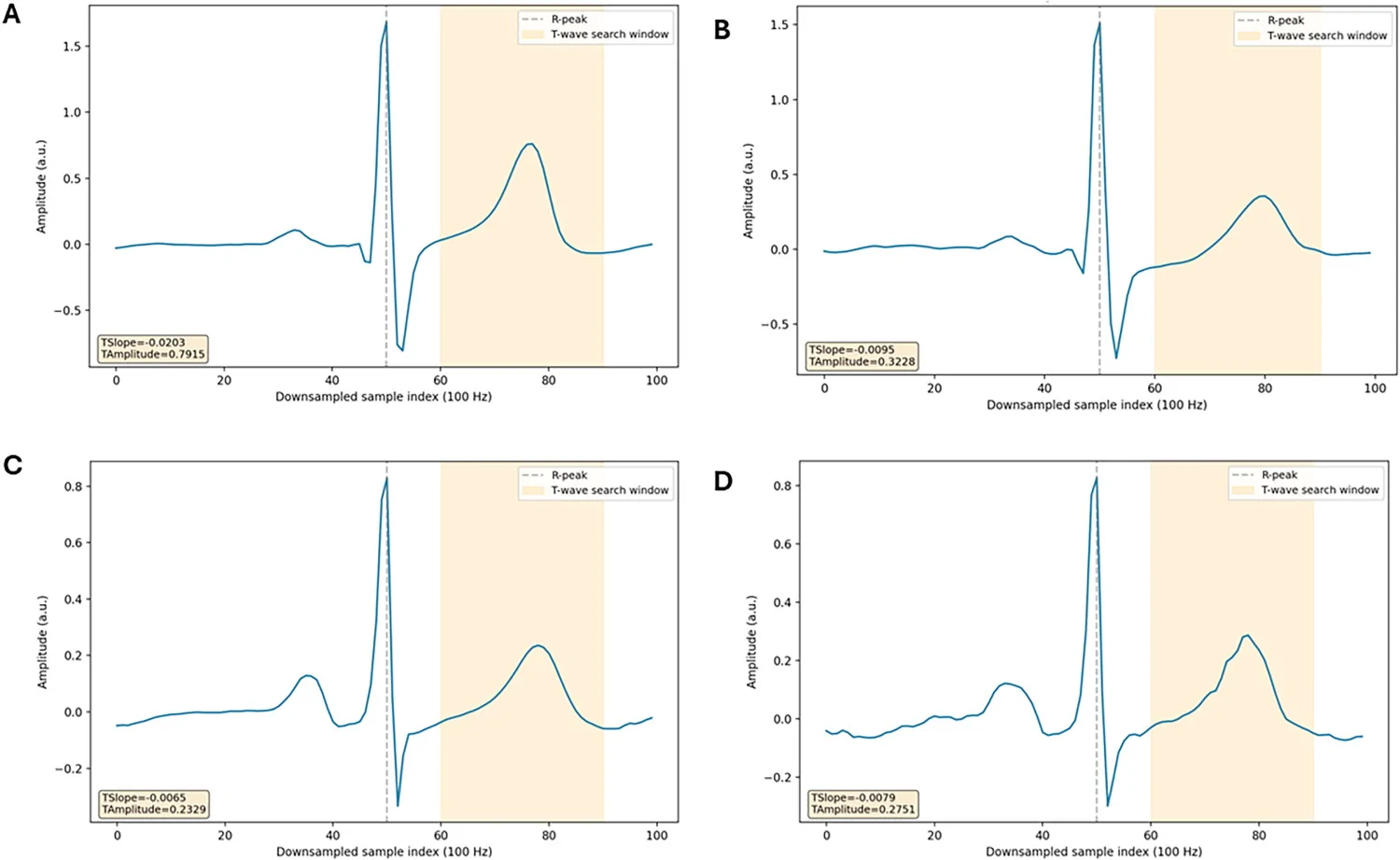
Representative single-lead ECG waveforms and extracted T-wave morphological features across clinical and classification states. Panel (A) and (B) Pre- vs. post-dialysis intradialytic dynamics: comparing paired recordings from the same session illustrates the physiological effect of potassium removal, showing clear T-wave amplitude attenuation and slope flattening as serum potassium drops from 4.70 to 3.20 mmol/L. Panel (C) and (D) Correct vs. incorrect classification mechanism: comparing correctly classified (potassium = 5.19 mmol/L) and misclassified (potassium = 3.10 mmol/L) cases from the same patient. In Panel (D), despite severe laboratory hypokalemia, the individual’s T-wave morphological features remained atypical (retaining a prominent amplitude, higher than the normokalemic trace in Panel (C)), directly demonstrating how atypical repolarization morphology drives model overestimation in certain hypokalemic states.

### Time-logarithmic potassium estimation

To overcome the inherent challenges of sparse biochemical sampling and enable precise, time-aligned training for our machine learning model, we implemented a novel time-logarithmic interpolation framework. During a standard HD session, serum potassium concentrations typically decrease in a distinct nonlinear pattern characterized by an initial rapid intradialytic decline, followed by a deceleration in clearance and an eventual plateau as the intracellular and extracellular compartments equilibrate [19,20]. To mathematically capture this kinetic behavior, we modeled the relationship between the elapsed dialysis time (*x*) and the normalized potassium concentration (*y*) using the following logarithmic function:

(1) y=loga(x+b)+cloga(b)+c

This approach provided continuous, high-resolution physiological labels, allowing the algorithm to map dynamic ECG features to precise interpolated potassium values at any given timestamp throughout the treatment session. A step-by-step mathematical derivation of this normalized time-logarithmic function, alongside a comparative evaluation of a model trained solely on discrete measured laboratory labels, is provided in the Supplemental Materials (Supplemental Figure S1).

### Training, testing, and comparison datasets

The clinical data were partitioned into three distinct datasets on the basis of chronological enrollment and sampling protocols (Figure 3). The algorithm training dataset comprised 45 patients who underwent MHD between June 2021 and December 2022, with central laboratory data collected biweekly during mid-week dialysis sessions. To capture the dynamic clearance of potassium, blood samples were obtained from the dialysis tubing at four specific intervals: Immediately prior to the initiation of HD, at 120 and 180 min into the session, and immediately upon treatment completion. An independent prospective testing dataset was subsequently established between January and December 2023 to evaluate the performance of the finalized machine learning algorithm, involving 29 consecutive patients who were entirely distinct from the training cohort and whose central laboratory data were collected monthly at two sampling points: Immediately before and immediately after the dialysis session. All 29 patients from the testing cohort were included in the comparison dataset and underwent additional POC potassium measurements at randomly selected time points aligned with the testing dataset sampling schedule, enabling head-to-head comparisons between the AI-ECG estimates and POC testing against the central laboratory standard.

**Figure 3.**
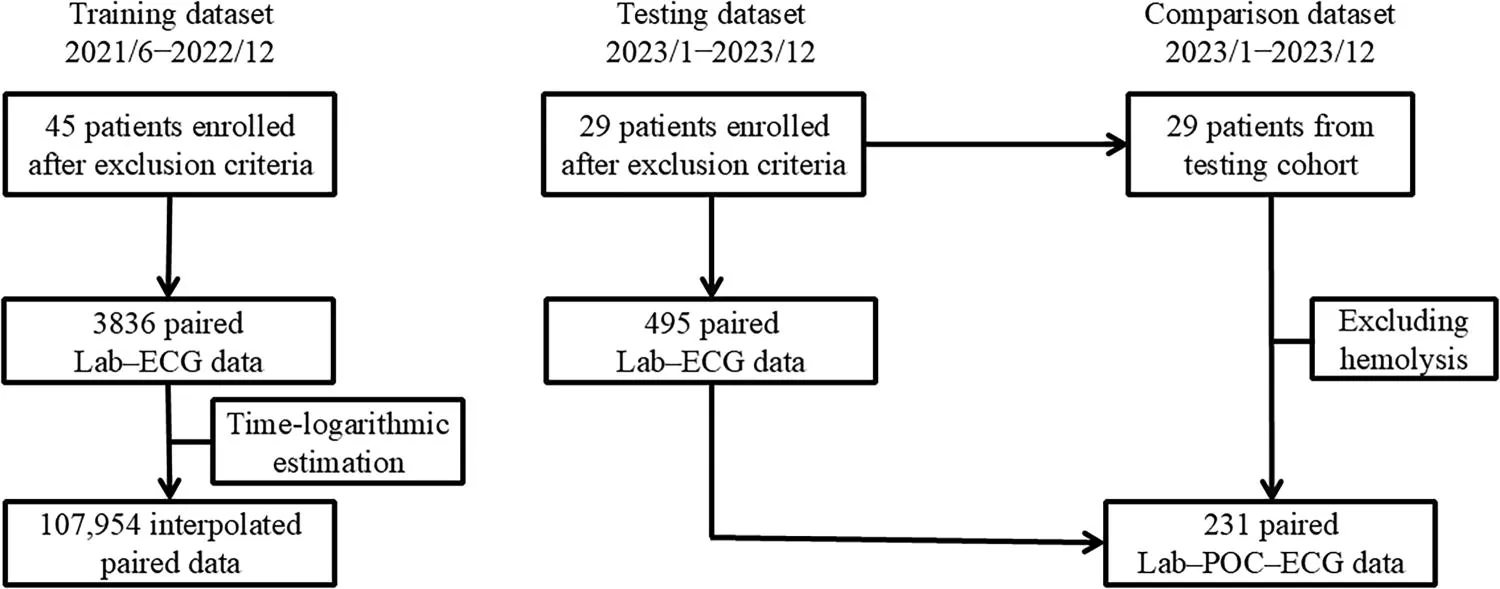
Study flowchart.

### Model development and implementation

The machine learning framework was implemented in Python (version 3.10.11) utilizing the Light Gradient Boosting Machine (LightGBM) architecture. LightGBM is an optimized gradient boosting framework based on decision trees that employs a leafwise tree growth strategy with strict depth constraints and histogram-based feature binning, yielding high computational efficiency and robust predictive performance. This specific algorithm was selected for our continuous ECG-derived potassium estimation framework because of its superior ability to capture intricate, nonlinear mathematical relationships and handle multidimensional physiological features without overfitting. Hyperparameters were optimized using the predefined training dataset through internal validation schemes, and the finalized model architecture was subsequently deployed to generate independent predictions on the testing dataset for final clinical validation. Details are presented in the Supplemental Materials.

### Statistical power evaluation

Statistical power was verified using PASS 2024 (NCSS, LLC, Kaysville, UT) for ROC curve analysis. The test evaluated a null hypothesis of AUC_0_ = 0.500 against an alternative hypothesis of AUC_1_ = 0.913 (derived from An et al. 2024) [21] at a two-sided *α* = 0.05. The primary statistical unit comprised 231 concurrent paired observations (88 hypokalemic and 143 non-hypokalemic events) across 29 prospective testing patients. To adjust for repeated intra-patient measurements (mean 8.0 draws/patient), intraclass correlation coefficients (ICC) were computed; using a conservative ICC = 0.262, the design effect (DEFF = 2.82) yielded a clustering-adjusted effective sample size of *N*_eff_ = 82 (31 hypokalemic events). Statistical power exceeded 99.9% under both raw (*N* = 231) and clustering-adjusted (*N*_eff_ = 82) sampling frameworks, consistent across discrete and continuous data-type specifications in PASS 2024.

### Statistical analysis

Statistical analyses were performed using R version 4.5.2, with receiver operating characteristic (ROC) curve analysis calculated using the pROC package version 1.18.5. Continuous variables were assessed for normality and are expressed as the mean ± standard deviation, while categorical variables are summarized as frequencies and percentages. Baseline characteristics were compared between groups using two-tailed unpaired Student’s *t* tests for continuous data and chi-square tests for categorical data. The predictive performance of the AI-ECG model was evaluated across both the regression and classification domains. For the continuous AI-ECG regression model, agreement with reference laboratory measurements was quantified using mean absolute error (MAE), Spearman’s rank correlation coefficient (*ρ*), and mean difference, visually illustrated by scatter plots and repeated-measures Bland–Altman analysis [22]. Diagnostic discrimination for hypokalemia screening was evaluated using AUC, derived directly from continuous outputs of the regression model. Diagnostic efficacy metrics—including sensitivity, specificity, positive predictive value, and negative predictive value (NPV)—were evaluated using the dedicated binary classification model. To account for repeated observations within patients across dialysis sessions, all reported confidence intervals for regression and diagnostic metrics, as well as pairwise comparisons between diagnostic AUC values, were estimated using patient-level cluster bootstrapping (2,000 resamples with replacement, clustered by patient identifier). The standard error, Z statistic, and 95% confidence interval (CI) for the between-method difference in AUC were derived directly from this cluster-bootstrap distribution. A two-sided *p* value <0.05 was considered to indicate statistical significance.

## Results

### Study cohort description and patient characteristics

A total of 282 HD sessions from 74 patients were analyzed, yielding an overall mean of 17.4 ± 10.1 treatments per patient; broken down by cohort, patients averaged 21.3 ± 10.8 treatments in the training dataset and 11.3 ± 5.1 treatments in the testing dataset. The prevalence of hypokalemia was 35.4, 32.3, and 41% in the training, testing, and comparison sets, respectively. Prior to dynamic curve fitting, the training dataset comprised 3,836 paired laboratory potassium–ECG measurements. By applying the normalized time-logarithmic potassium estimation framework, we successfully generated a high-density training matrix containing 107,954 time-aligned, paired potassium–ECG data points. The independent prospective testing dataset comprised 495 paired measurements. Detailed study flow is presented in Supplemental Materials Table S1. As summarized in Table 1, no significant baseline demographic or clinical differences were observed between the training and testing cohorts.

**Table 1.** Baseline characteristics of the study participants.

Parameters	Training cohort	Testing cohort	*p*
N	45	29	
Sex (% male)	53.3	62.1%	0.459
Age (years)	59.8 ± 11.4	59.8 ± 12.9	0.990
Height (cm)	162.5 ± 8.3	163.6 ± 8.0	0.579
Weight (kg)	65.9 ± 12.8	66.8 ± 14.2	0.798
BMI (kg/m^2^)	24.8 ± 3.5	24.8 ± 4.2	0.984
Underlying comorbidities			
Hypertension (%)	88.9	82.8	0.451
Diabetes mellitus (%)	51.1	55.2	0.733
Coronary artery disease (%)	46.7	44.8	0.877
Heart failure (%)	22.2	24.1	0.848
Laboratory data			
Kt/V	1.65 ± 0.29	1.60 ± 0.27	0.486
Hemoglobin (g/dL)	10.7 ± 1.1	11.1 ± 1.0	0.188
BUN (mg/dL)	67.9 ± 19.9	70.1 ± 13.4	0.596
Creatinine (mg/dL)	10.6 ± 1.9	10.6 ± 2.0	0.942
Potassium (mmol/L)	4.5 ± 0.6	4.5 ± 0.6	0.995
Total calcium (mg/dL)	9.4 ± 0.9	9.2 ± 1.1	0.334
Phosphate (mg/dL)	5.3 ± 1.4	5.4 ± 1.0	0.710
Albumin (g/dL)	4.0 ± 0.2	3.9 ± 0.2	0.559

Abbreviations: BMI, body mass index; BUN, blood urea nitrogen.

### Performance of the ECG model in the testing cohort

In the full prospective testing cohort, the AI-ECG regression model demonstrated moderate overall continuous agreement with central laboratory reference standards, yielding a mean difference of 0.417 mmol/L (95% CI: 0.293–0.541 mmol/L) and 95% limits of agreement ranging from −0.871 to 1.704 mmol/L. Continuous prediction errors varied substantially across potassium concentrations, with markedly higher discrepancies during absolute hypokalemia (MAE = 1.078 mmol/L) than non-hypokalemia (MAE = 0.401 mmol/L). For binary hypokalemia screening, continuous predictions from the AI-ECG regression model demonstrated moderate diagnostic discrimination, achieving an AUC of 0.713 (95% CI: 0.618 − 0.799). In parallel, the dedicated AI-ECG classification model achieved a sensitivity of 78.5% (95% CI: 67.1 − 88.2%) and a clinically reassuring NPV of 86.8% (95% CI: 79.8–92.9%) at an operating specificity of 67.6% (95 CI: 53.2 − 81.1%).

### Head-to-head comparison with POC testing

To ensure a rigorous head-to-head evaluation, direct comparative analyses between AI-ECG, POC test, and central laboratory reference measurements were restricted to identical concurrent time points (laboratory–POC–ECG triplets). Out of 495 total laboratory–ECG pairs in the testing cohort, 231 time points contained concurrent POC measurements (88 hypokalemic events, 38.1%). Within this concurrent triplet subset, continuous potassium predictions from the AI-ECG regression model yielded a mean difference of 0.454 mmol/L (95% limits of agreement −0.840 to 1.748 mmol/L) relative to central laboratory standards (Table 2 and Figures 4 and 5). Although the predictive variance increased during absolute hypokalemic states (MAE of 1.000 mmol/L), the precision of the algorithm increased within the non-hypokalemic range (MAE of 0.479 mmol/L). The regression model exhibited significant negative proportional bias (slope −0.773, 95% CI: −0.831 to −0.718; *p* < 0.001), overestimating serum potassium by 1.111 mmol/L at a laboratory standard of 3.0 mmol/L and underestimating it by 0.434 mmol/L at 5.0 mmol/L. Sensitivity analysis using pairwise mean potassium as the abscissa confirmed this directional bias, yielding a slope of −0.928 (95% CI: −1.049 to −0.811). In contrast, POC measurements demonstrated the tightest concordance with central laboratory values, with a mean difference of 0.179 mmol/L (95% limits of agreement −0.115 to 0.474 mmol/L), a strong correlation (ρ = 0.977), and an overall MAE of 0.201 mmol/L. For binary hypokalemia screening, continuous estimates derived from the AI-ECG regression model achieved moderate diagnostic discrimination, yielding an AUC of 0.722 (95% CI: 0.639–0.795). In parallel, the dedicated AI-ECG binary classification model retained a sensitivity of 77.9% alongside a clinically reassuring NPV. As expected, the POC test exhibited near-perfect diagnostic discrimination (AUC 0.996), significantly outperforming the noninvasive AI-ECG model (ΔAUC = 0.274, 95% CI: 0.200–0.356, *Z* = 6.74, *p* < 0.0001).

**Figure 4.**
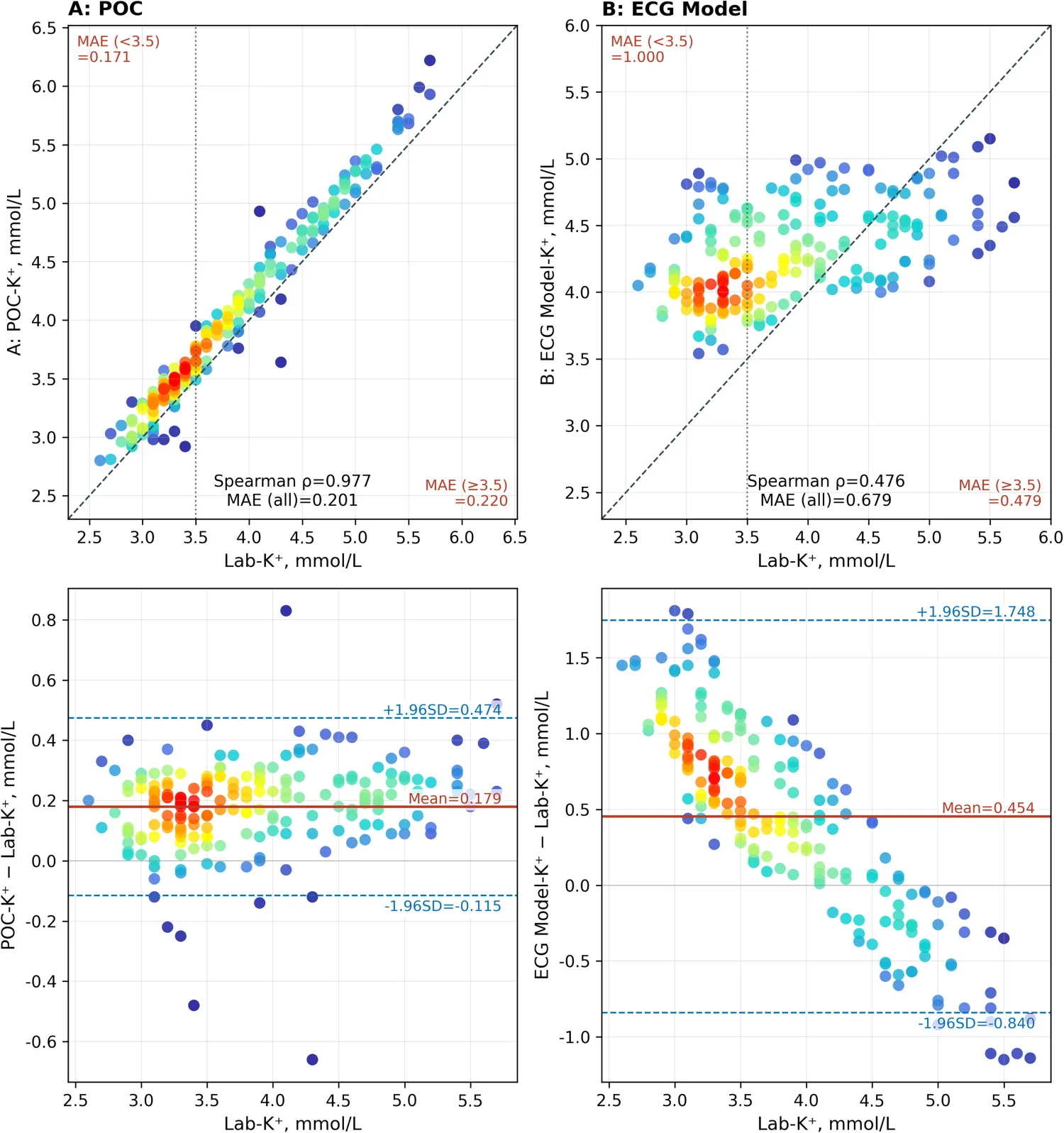
Scatter plots and Bland-Altman plots of POC test and ECG model. ECG, electrocardiography; POC, point-of-care.

**Figure 5.**
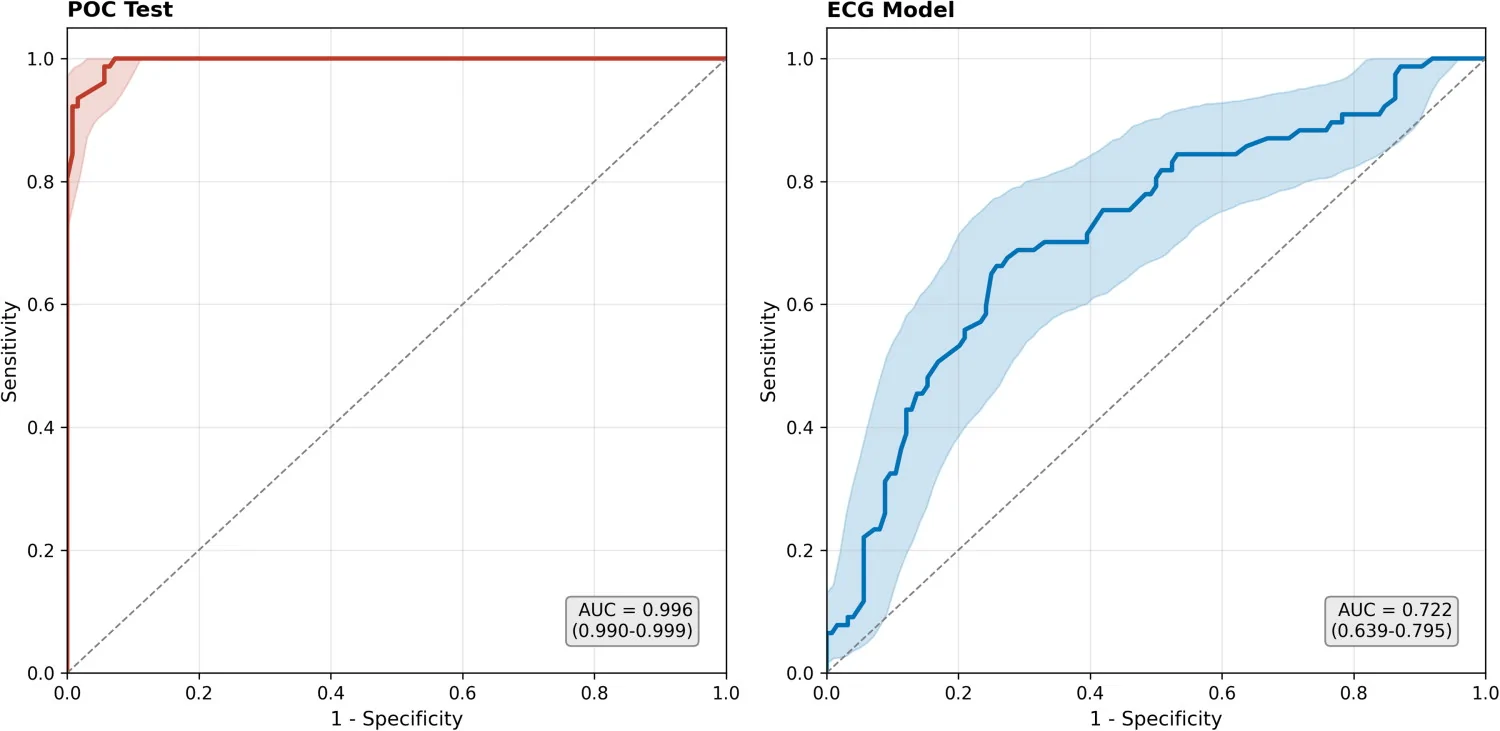
ROC curves of the POC test and ECG model. AUC, area under the receiver operating characteristic curve; ECG, electrocardiography; POC, point-of-care; ROC, receiver operating characteristic curve.

**Table 2.** Diagnostic performance of the POC test and ECG model for hypokalemia detection.

Variable	POC test	ECG regression model	ECG classification model
No. of observations	231	231	231
No. of patients	29	29	29
No. of hypokalemic observations	88 (38.1%)	88 (38.1%)	88 (38.1%)
MAE (mmol/L) (all)	0.201	0.679	N/A
MAE (mmol/L) (K+ <3.5 mmol/L)	0.171	1.000	N/A
MAE (mmol/L) (K+ ≥3.5 mmol/L)	0.220	0.479	N/A
Spearman correlation (ρ)	0.977	0.476	N/A
Mean difference (mmol/L) (mean ± SD)	0.179 ± 0.150	0.454 ± 0.660	N/A
Mean difference (mmol/L) (mean, 95% CI)	0.179 (0.148–0.211)	0.454 (0.319–0.588)	N/A
95% LoA upper (95% CI)	0.474 (0.418–0.524)	1.748 (1.601–1.869)	N/A
95% LoA lower (95% CI)	−0.115 (−0.185 to −0.037)	−0.840 (−1.041 to −0.600)	N/A
Within-subject SD of differences (mmol/L)	0.137	0.609	N/A
Between-subject SD of differences (mmol/L)	0.063	0.256	N/A
AUC (95% CI)	0.996 (0.990–0.999)	0.722 (0.639–0.795)	N/A
Sensitivity (%) (95% CI)	98.7 (95.4–100.0)	66.2 (51.2–79.6)	77.9 (68.7–85.4)
Specificity (%) (95% CI)	94.4 (90.5–97.7)	74.2 (65.5–82.4)	67.7 (58.5–77.1)
PPV (%) (95% CI)	91.6 (85.1–96.7)	61.4 (47.2–74.1)	60.0 (45.9–73.3)
NPV (%) (95% CI)	99.2 (97.1–100.0)	78.0 (71.1–85.6)	83.2 (77.3–88.8)
Accuracy (%) (95% CI)	96.0 (93.4–98.1)	71.1 (65.3–76.6)	71.6 (64.8–77.7)

Abbreviations: AUC, area under the receiver operating characteristic curve; CI, confidence interval; ECG, electrocardiography; LoA, limits of agreement; MAE, mean absolute error; NPV, negative predictive value; POC, point of care; PPV, positive predictive value; SD, standard deviation.

Note.

(1) All diagnostic and continuous metrics across all columns were evaluated on the identical set of *N* = 231 concurrent laboratory–POC–ECG triplet observations (88 hypokalemic events, 38.1%) obtained across 29 prospective testing patients.

(2) The AI-ECG framework incorporates two distinct models trained on identical ECG features: a continuous regression model predicting serum potassium concentration and a dedicated classification model predicting binary hypokalemia. Because these models operate independently, their respective performance metrics are reported in separate columns.

(3) All reported 95% confidence intervals reflect patient-level cluster bootstrapping (2,000 resamples with replacement) to account for repeated intra-patient measurements.

## Discussion

In this study of patients with ESRD receiving MHD, our single-lead AI-ECG model demonstrated clinically meaningful diagnostic value for noninvasive serum potassium estimation and hypokalemia screening. Although the algorithm’s predictive precision was optimized within the non-hypokalemic range, it maintained a high sensitivity (77.9%) and a robust NPV (83.2%) for identifying hypokalemia. These metrics highlight its preliminary utility as a noninvasive screening tool in a population highly vulnerable to rapid electrolyte shifts. Although predictive variance increased during hypokalemic states (potassium < 3.5 mmol/L), the algorithm’s diagnostic performance suggests potential for adjunctive physiological surveillance during HD. As expected, compared with the AI model, POC testing maintained technical superiority in terms of the MAE and tight laboratory concordance. Nevertheless, the noninvasive AI-ECG approach offers a promising, bloodless framework for physiological trend monitoring, providing a potential adjunctive strategy to complement intermittent testing.

AI-enabled models trained on standard 12-lead ECG have demonstrated outstanding diagnostic performance in estimating serum potassium concentrations. For instance, the ECG12Net algorithm, trained on more than 66,000 ECGs, achieved an AUC of 0.75 and a 50.7% sensitivity for general hypokalemia screening; notably, its accuracy increased to an AUC of 0.976, with sensitivities exceeding 95% for detecting severe hypokalemia (potassium concentration ≦2.5 mmol/L) [13]. Subsequent real-world evaluations in more than 30,000 emergency department patients confirmed this robust performance, with an AUC range of 0.73–0.76, sensitivities between 73.8% and 76.9%, and NPVs exceeding 90%, with accuracy scaling up to an AUC of 0.95 and a sensitivity of 93.3% during critical hypokalemic states [23]. Furthermore, a multicenter analysis encompassing approximately 480,000 ECG–potassium pairs generated an external validation AUC above 0.91, with a sensitivity over 79% for hypokalemia identification [21]. Despite these impressive predictive capabilities, the translation of 12-lead AI frameworks into routine HD care is bottlenecked by structural constraints. Continuous monitoring during multihour treatment sessions using a full 12-lead apparatus is logistically challenging, highly labor intensive for clinical staff, and physically restrictive for the patient. Consequently, there is an urgent clinical imperative for simplified, single-lead monitoring systems that successfully balance diagnostic accuracy with real-time operational feasibility at the bedside.

Single-lead and limited-lead ECG systems offer distinct advantages for the HD environment because they are noninvasive, simple to deploy, and well suited for continuous monitoring. While these systems inherently provide less spatial resolution than standard 12-lead recordings do, their clinical utility is increasingly supported by evidence. For instance, limited-lead ECGs have demonstrated the ability to detect hyperkalemia with clinically meaningful accuracy [24,25]. Furthermore, research comparing single-lead smartwatches to 12-lead ECGs revealed only minimal differences in potassium monitoring accuracy [26]. In specific HD cohorts, high-resolution single-lead recordings paired with advanced signal processing yielded MAEs of 0.36 ± 0.34 mmol/L for personalized models and 0.5 ± 0.42 for global models [14]. A large multicenter analysis further underscores this potential, reporting an AUC exceeding 0.87 for single-lead hypokalemia detection, with sensitivity, specificity, and NPV values exceeding 79.4, 78.9, and 98.5%, respectively [21]. Our model directly aligns with this paradigm, maintaining a solid discriminatory capacity (AUC 0.722) and a comparable sensitivity profile (77.9%) for acute hypokalemic events. The relatively modest specificity (67.7%) observed in our cohort likely reflects the intricate cardiovascular phenotypes common among patients receiving MHD—such as advanced structural heart disease, left ventricular hypertrophy, and chronic intracellular electrolyte adaptations—which can attenuate or confound subtle, potassium-dependent repolarization changes in a single-lead vector.

Translating these noninvasive monitoring models into routine clinical workflows requires addressing critical real-world signal acquisition challenges, including patient movement, sweat-induced lead loosening, and electromagnetic interference from blood pumps. Beyond algorithmic refinement, the fidelity of continuous physiological tracking depends heavily on mechanical interface stability, sensor placement, and acquisition quality in clinical- or consumer-grade sensing architectures. Recent engineering advances in noninvasive cardiac signal acquisition—such as phonocardiographic and mechanical biosensing modalities—highlight that physical sensor coupling and positioning are primary determinants of signal-to-noise ratio and artifact resilience during real-time deployment [27]. In the context of intradialytic monitoring, ensuring robust physical sensor adhesion and standardized lead placement remains equally vital to prevent motion-induced baseline wander from confounding fine electrophysiological features such as T-wave morphology.

POC potassium testing is essential when rapid electrolyte assessment is needed to guide immediate clinical interventions, particularly in high-acuity settings such as emergency departments, intensive care units, and HD units [8,10,28]. Extensive literature has established that POC platforms provide robust analytical agreement with central laboratory standards. In populations with chronic kidney disease, prior investigations have reported excellent concordance with laboratory assays, with ICC greater than 0.90 and remarkably small mean bias values typically ranging between 0.39 and 0.43 mmol/L [28,29]. Furthermore, a large-scale multicenter evaluation confirmed the robust diagnostic utility of POC systems, achieving an AUC of 0.933 for dyskalemia screening [30]. In our cohort, compared with these established historical benchmarks, the POC device exhibited even stronger agreement with laboratory measurements (MAE 0.201 mmol/L) [30]. Its diagnostic performance for the classification of hypokalemia was exceptional (AUC 0.996, sensitivity 98.7%), reinforcing its role as a reliable alternative to conventional laboratory assays at the bedside. Although POC testing is susceptible to preanalytical variability from hemolysis or sample handling, its precision in the current study underscore its clinical reliability during active renal replacement therapy [9,31].

The superior accuracy of POC testing compared with that of the AI-ECG model is fundamentally expected, as POC systems directly quantify whole-blood biochemistry, whereas the machine learning model indirectly estimates potassium *via* electrophysiological changes. This indirect approach is inherently more susceptible to confounding factors prevalent in patients receiving MHD. Furthermore, as noted in previous AI-ECG studies, detecting mild-to-moderate hypokalemia (2.5< potassium concentration ≤3.5 mmol/L) is challenging because the associated T-wave flattening can be extremely subtle and overlap with normal morphological variations [13,30]. In our study, the continuous regression model systematically overestimates potassium concentrations in the hypokalemic range while underestimating hyperkalemic levels. Clinically, overestimation during true hypokalemia poses a critical safety concern, as falsely elevated predictions could provide unwarranted reassurance and delay confirmatory blood draws or timely electrolyte replacement. From a translational standpoint, however, these two modalities present highly divergent, complementary strengths. POC systems involve significant recurring costs for consumables and require staff time for blood sampling, making frequent measurements resource intensive [32]. In contrast, AI-enabled ECG analysis offers an economically scalable framework for automated physiological monitoring [30]. Consequently, rather than viewing these technologies as competing alternatives, clinical workflows should integrate them into a hierarchical safety protocol: utilizing dedicated binary classification algorithms optimized for high sensitivity to flag potential events, treating continuous AI-ECG as an adjunctive trend-monitoring tool between scheduled draws, and mandating POC or central laboratory confirmation prior to therapeutic intervention.

The primary strength of this investigation lies in its novelty as the first head-to-head diagnostic comparison evaluating both POC testing and AI-ECG estimation against a central laboratory reference in the MHD population. However, this study had several limitations warrant careful consideration, First, it was a single-center study with a modest sample size, which may limit its generalizability. Second, ECG-derived potassium estimation is inherently susceptible to confounding from comorbidities common in dialysis patients, which may reduce specificity. Third, dialysate potassium concentrations were not recorded in our observational protocol. The serum-to-dialysate potassium gradient is a primary determinant of myocardial potassium flux, where steep gradients accelerate repolarization kinetics, drive rapid T-wave morphological changes, and heighten arrhythmogenic risk. Fourth, our feature engineering focused on T-wave morphology rather than dynamic QTc tracking. Automated QT delineation during continuous intradialytic monitoring remains technically challenging due to single-lead noise and hypokalemia-induced T–U wave fusion. Integrating dynamic QTc shifts alongside repolarization morphology warrants future investigation to capture potential synergistic diagnostic signals. Fifth, although our model incorporated temporal fitting for potassium estimation, external validation across diverse dialysis settings is needed before broader implementation. Sixth, reference potassium values in our testing cohort were obtained exclusively at pre-dialysis and post-dialysis timepoints. Consequently, the analytical precision of continuous model predictions during mid-session trajectory transitions and rapid electrolyte shifts was not independently validated against concurrent blood draws. Finally, owing to the very low prevalence of severe hypokalemia (potassium concentration ˂2.5 mmol/L) within our study cohort, we were unable to evaluate the diagnostic performance of the ECG model in this extreme range. While severe hypokalemia rarely occurs during and immediately following standard HD procedures, future prospective multicenter trials incorporating serial intra-session laboratory sampling are needed to formally establish mid-session tracking fidelity and validate the algorithm’s sensitivity across the full spectrum of severe dyskalemia.

## Conclusions

Our AI-enabled single-lead ECG model demonstrates preliminary screening potential for intradialytic hypokalemia alongside reasonable estimation performance during normokalemia. While central laboratory assays and POC testing remain indispensable for definitive analytical precision and acute clinical decision-making, this AI-ECG approach provides a noninvasive framework for intradialytic trend monitoring. Future prospective multicenter trials incorporating intra-session sampling and clinical decision-impact evaluations are needed to confirm the generalizability and true clinical utility of noninvasive electrolyte monitoring in MHD.

## Supplementary Material

Supplemental Materials.docx

## Data Availability

The participants of this study did not give written consent for their data to be shared publicly, so due to the sensitive nature of the research supporting data is not available.
