# Supplementary material for: Comparison of an AI-enabled single-lead ECG monitor and point-of-care testing for detecting intradialytic hypokalemia
Source: Ren Fail. 2026 Oct 30;48(1):2746815. doi: 10.1080/0886022X.2026.2746815 (PMC13726304; doi:10.1080/0886022X.2026.2746815)
Supplement: Supplementary file 1 [file IRNF_A_2746815_SM0470.docx]

**Comparison of an AI-Enabled Single-Lead ECG Monitor and Point-of-Care Testing for Detecting Intradialytic Hypokalemia**

**Supplemental Materials**

**Table of Contents:**

1. **Methods**
(1) **ECG Preprocessing and Feature Extraction**
(2) **Model Development and Validation**
(3) **Generation of the continuous potassium reference trajectory via time-logarithmic curve fitting**
(4) **Supplemental Figure S1.** Generation of the continuous potassium reference trajectory via time-logarithmic curve fitting.
(5) **Construction of Equation (1)**
(6) **Handling missing or incomplete sampling points**
(7) **Evaluation of interpolation accuracy against withheld laboratory measurements**
(8) **Comparison with a model trained only on measured laboratory labels**
2. **Results**
(1) **Supplemental Table S1.** Study flow in the training and testing cohorts
(2) **Subgroup analysis** **in patients with cardiovascular comorbidities**

**Methods**

**ECG Preprocessing and Feature Extraction**

1. Signal acquisition, filtering, and artifact rejection
(1) ECG sampling frequency

Single-lead ECG signals were continuously recorded at a sampling frequency of 500 Hz using an IntelliVue MP5 monitor.

(2) Numerical filter specifications, powerline-removal procedure, and wavelet/decomposition settings

Denoising was performed in two sequential stages:

- Stage 1 (wavelet decomposition & frequency selection): signals were decomposed via Discrete Wavelet Transform (biorthogonal wavelet "bior4.4", 9 decomposition levels). The lowest-frequency approximation coefficient (corresponding to frequencies below approximately 0.49 Hz, i.e., baseline wander) and the three highest-frequency detail coefficients (corresponding to approximately 31.25–250 Hz) were zeroed, and the signal was reconstructed via the inverse wavelet transform, effectively retaining a passband of approximately 0.49–31.25 Hz. Fixed-frequency powerline interference (50/60 Hz) falls above this retained passband and was eliminated through this frequency selection without requiring a separate notch filter.
- Stage 2 (baseline wander suppression): residual baseline wander was further suppressed by subtracting the output of a cascaded median filter (window widths of 0.2 s and 0.6 s) from the wavelet-filtered signal.

(3) R-peak detection method

R-peaks were identified using the processing pipeline in the biosppy Python library, which applies an internal 0.67–45 Hz FIR bandpass filter, the Hamilton segmentation algorithm for peak localization, and the library’s built-in R-peak correction routine (tolerance = 0.05 s).

(4) Quantitative artifact-rejection criteria

Individual beat waveforms were excluded if the maximum amplitude exceeded a fixed positive threshold (saturation or gross artifact), the minimum amplitude fell below the corresponding negative threshold, or the maximum amplitude fell below a minimal-signal floor (flatline or undetectable waveform).

2. Complete feature set

For each detected R-peak, a window of 250 samples before and 250 samples after the peak was extracted, ensemble-averaged across consecutive beats to reduce noise, and downsampled to 100 Hz to form a 100-point waveform. The final model input comprised this 100-point waveform concatenated with two T-wave morphological features (T-wave slope and T-wave amplitude), motivated by prior literature establishing their association with serum potassium concentration.^1^

**Model Development and Validation**

1. LightGBM hyperparameters, internal validation, and model selection
(1) LightGBM hyperparameters

Model configuration relied on default library settings: boosting type = gbdt, number of leaves = 31, maximum tree depth = unlimited, learning rate = 0.1, number of estimators = 100, minimum child samples = 20, subsample ratio = 1.0, column-sample ratio = 1.0, L1/L2 regularization = 0. Only the random seed was fixed for reproducibility; no exhaustive hyperparameter search was performed.

(2) Internal validation procedure

Hyperparameter evaluation and model comparisons within the training cohort were conducted using patient-grouped *k*-fold cross-validation, ensuring that no single patient’s data appeared in both the training and validation folds of any split.

(3) Model selection criterion

Candidate regression models (including linear regression and LightGBM) were evaluated based on patient-grouped cross-validation MAE on the training set. LightGBM was selected for its optimal balance of predictive accuracy and computational efficiency derived from leaf-wise tree growth and histogram-based feature binning

(4) Confirmation of patient/session-level internal validation

The training (*n* = 45) and testing (*n* = 29) cohorts consisted of entirely distinct patients with zero overlap. Cross-validation within the training cohort was strictly grouped by patient ID, confirming that correlated ECG windows from the same patient were never split across training and validation folds.

2. Regression vs. classification pipeline and threshold selection
(1) Separate models for regression and classification

Regression (continuous potassium estimation) and hypokalemia classification were implemented as two independent models rather than deriving one output from the other.

(2) Classification threshold selection

The dedicated binary LightGBM classifier categorized observations using a fixed, default probability decision threshold of 0.5 (predicted probability ≥ 0.5 classified as positive). This was a predetermined default threshold and was neither tuned nor adjusted using the test set.

**Generation of the continuous potassium reference trajectory via time-logarithmic curve fitting**

To establish a high-resolution, continuous ground truth for the ECG-based prediction model, we employed a time-logarithmic curve-fitting approach. Prior studies have modeled potassium kinetics during hemodialysis using compartmental and nonlinear kinetic models.^2,3^ The time-logarithmic model used in this study was a data-driven empirical approximation designed to interpolate the nonlinear temporal trajectory of serum potassium rather than serve as a mechanistic physiological model. This process yielded a smooth, continuous reference curve applied across hemodialysis sessions, representing the estimated temporal trajectory of potassium clearance. These continuous trajectories supplied the dense target labels required to train the ECG-based algorithm, mapping cardiac electrical features to precise, interpolated potassium concentrations between the physical blood sampling intervals.

**Supplemental Figure S1.** Generation of the continuous potassium reference trajectory via time-logarithmic curve fitting. Hourly serum potassium measurements were normalized to a 0–1 scale and are represented as discrete data points (blue dots). These points were used to optimize the parameters of the logarithmic model (Equation 1) via nonlinear regression, resulting in a continuous, shared reference curve (red line) applied across sessions.

**Equation (1) is constructed as follows:**

1. $y=\log_{a}(x)$:
   The model is based on the logarithmic form $y=\log_{a}(x)$, where the base *a* serves as the curvature parameter. This parameter dictates the nonlinearity of the function; it is specifically chosen to model the rapid-onset, slow-plateau" behavior characteristic of solute diffusion. In this context, *a* influences the rate at which the initial steep decline transitions into a gradual flattening over the course of the dialysis session.
2. $y=\log_{a}(b+x)+c$:
   To ensure that the model can be fitted to real-world clinical data, horizontal and vertical offsets were introduced:
   - Horizontal offset (*b*): The parameter *b* shifts the curve along the *x*-axis. This ensures that the argument of the logarithm remains positive at *t* = 0 and allows the model to account for the initial equilibration delay often observed at the start of hemodialysis.
   - Vertical offset (*c*): The parameter *c* provides a vertical translation. This allows the function to align with the specific magnitude of the measured potassium concentrations and the patient’s asymptotic baseline.
3. To create a standardized reference for the machine learning model, the expression was normalized by its value at *x* = 0. This yielded the final governing equation:

$y=\frac{\log_{a}(b+x)+c}{\log_{a}(b)+c}$:
This normalization establishes a critical boundary condition where *y* = 1 at *x* = 0, representing the 100% baseline potassium concentration for every patient. By dividing by the term $y=\log_{a}(b)+c$, the model focuses on the relative rate of decay rather than absolute values. Parameters *a*, *b*, and *c* were estimated globally once from the pooled training cohort. Specifically, for every dialysis session in the training set containing a complete set of four measured potassium–ECG timepoints, elapsed time was zero-referenced to the initial sampling time (*t* = 0), and serum potassium concentrations were min–max normalized to a [0, 1] relative scale within that session. These normalized (time, potassium) coordinates from all qualifying sessions were pooled into a single dataset. Parameters *a*, *b*, and *c* were then jointly optimized via a Bayesian hyperparameter search to minimize the squared error between the fitted curve and the pooled normalized points, ensuring the curve captures the shared non-linear trajectory common across sessions. The resulting fixed values were subsequently applied uniformly to every dialysis session requiring interpolation.

4. Conversion of normalized equation to absolute potassium concentration

For a given session, the normalized curve value *y*(*t*) obtained from Equation (1) was denormalized to an absolute potassium concentration *K*(*t*) using that specific session’s measured potassium values at its first (*K*_first_) and last (*K*_last_) sampling points:

*K*(*t*) = *y*(*t*) × (*K*_first_ − *K*_last_) + *K*_last_

**Handling missing or incomplete sampling points**

When a session had all four scheduled measurements, potassium concentrations between consecutive measured points were derived using direct piecewise-linear interpolation, and Equation (1) was not required. When a session contained only two or three successfully obtained measurements, the time-logarithmic curve was applied instead, anchored to that session's first and last available measurements. Sessions with fewer than two valid measurements or more than four were excluded entirely from the interpolation pipeline.

**Evaluation of interpolation accuracy against withheld laboratory measurements**

Because parameters *a*, *b*, and *c* were fitted using exclusively sessions with four complete measurements, sessions with three measurements provided a clean, held-out validation set. In a three-draw session, the middle (second) measurement is completely independent: it was omitted from the parameter fitting pool and was not used to anchor the session's denormalization (only the first and last measurements were used). We compared the curve's predicted value at the timestamp of the middle measurement against this withheld laboratory value. The interpolation method achieved a mean absolute error of 0.111 mmol/L (SD = 0.071, median = 0.111 mmol/L). Because this interpolation error is substantially smaller than the overall ECG model error, these results demonstrate that the time-logarithmic curve provides highly accurate reference labels, indicating that downstream model prediction error stems primarily from the ECG-to-potassium estimation step rather than label interpolation quality.

**Comparison with a model trained only on measured laboratory labels**

We performed a direct comparison using patient-grouped cross-validation. For the same set of patients, one model was trained using exclusively directly measured laboratory–ECG pairs, and a second model was trained using exclusively interpolated pairs. Both models were then evaluated on the measured laboratory values of entirely held-out patients who contributed no data to either training set. Classification performance was virtually identical between the two approaches (accuracy 57.0% vs. 56.9%; AUC 0.607 vs. 0.600). For regression, the interpolation-trained model achieved a lower prediction error (MAE 0.495 mmol/L vs. 0.544 mmol/L). We attribute this improvement in regression accuracy to the increase in training sample size, which outweighed the modest labeling noise introduced by interpolation. Together, these results demonstrate that interpolation-based data augmentation improves regression optimization without compromising performance.

**Results**

**Supplemental Table S1.** Study flow in the training and testing cohorts

| **Study Flow Parameter** | **Training Cohort** | **Testing Cohort** | **Total Cohort** |
| --- | --- | --- | --- |
| **Enrolled patients** | 45 | 29 | 74 |
| **Concurrent clinical shifts** | 202 | 80 | 282 |
| **Individual patient-treatments** | 959 | 329 | 1288 |
| **Measured laboratory samples** | 4036 | 628 | 4664 |
| **Valid ECG segments** | 3929 | 530 | 4459 |
| **Excluded ECG segments** | 307 | 128 | 435 |
| **Interpolated training labels** | 107954 | N/A | 107954 |
| **Hypokalemic observations** | 1428 | 203 | 1631 |
| **Observations included in final analysis** | 3836 | 495 | 4331 |

**Subgroup analysis** **in patients with cardiovascular comorbidities**

Because patients on hemodialysis with underlying cardiovascular comorbidities—specifically coronary artery disease and heart failure—are at heightened risk for intradialytic hypokalemia–induced arrhythmias, we performed a dedicated subgroup retraining and validation analysis. The cardiovascular retraining cohort comprised 23 patients with 2,163 laboratory blood draws and 2,109 quality-filtered ECG recordings. Application of the time-logarithmic interpolation framework generated 56,167 continuous training labels, establishing a final training set of 1,996 Lab–ECG paired observations (760 hypokalemic events, 38.1%). The independent cardiovascular testing cohort included 18 distinct patients (391 laboratory blood draws and 331 filtered ECGs), yielding 318 Lab–ECG paired observations for prospective evaluation (129 hypokalemic events, 40.6%).When evaluated on the 18 cardiovascular testing patients, the continuous AI-ECG regression model demonstrated poor predictive tracking, yielding a MAE of 0.701 mmol/L, a negligible rank correlation with laboratory standards (*ρ* = −0.012), and a mean difference of 0.141 mmol/L (95% CI: −0.074 to 0.357 mmol/L) with wide repeated-measures limits of agreement (−1.464 to 1.747 mmol/L). Furthermore, the continuous regression estimates failed to show discriminative ability for hypokalemia screening (AUC = 0.414, 95% CI: 0.309−0.519). In contrast, the dedicated retrained AI-ECG binary classification model achieved moderate diagnostic utility on these same testing observations, yielding an overall accuracy of 71.1%, a sensitivity of 62.2% (95% CI: 42.0−79.2%), and a specificity of 77.3% (95% CI: 64.5−89.1%). Notably, performance in this retrained cardiovascular subgroup did not exceed that of the primary overall cohort. Compared to the overall testing population, continuous regression tracking in the cardiovascular subgroup exhibited diminished predictive accuracy (MAE: 0.701 vs. 0.620 mmol/L; AUC: 0.414 vs. 0.713), while the dedicated binary classifier achieved comparable overall accuracy (71.1 vs. 71.1%) but lower sensitivity (62.2% vs. 78.5%). Retraining specifically in patients with cardiovascular disease did not outperform the primary model, indicating that underlying structural heart disease introduces repolarization heterogeneity that complicates single-lead T-wave feature tracking.

**References**

1. Attia ZI, DeSimone CV, Dillon JJ, et al. Novel bloodless potassium determination using a signal-processed single-lead ECG. J Am Heart Assoc. 2016;5(1):e002746.

2.Agar BU, Culleton BF, Fluck R, Leypoldt JK. Potassium kinetics during hemodialysis. Hemodial Int. 2015;19(1):23-32.

3.Ursino M, Donati G. Mathematical model of potassium profiling in chronic dialysis. Contrib Nephrol. 2017;190:134-145.
